# A new inversion method of estimation of simultaneous near surface bulk density variations and terrain correction across the Bandar Charak (Hormozgan-Iran)

**DOI:** 10.1186/2193-1801-3-135

**Published:** 2014-03-10

**Authors:** Reza Toushmalani, Azizalah Rahmati

**Affiliations:** Department of Computer, Faculty of engineering, Islamic Azad University, Kangavar Branch, Kangavar, Iran

**Keywords:** Inversion method, Estimation near surface bulk density variations, Terrain correction

## Abstract

A gravity inversion method based on the Nettleton-Parasnis technique is used to estimate near surface density in an area without exposed outcrop or where outcrop occurrences do not adequately represent the subsurface rock densities. Its accuracy, however, strongly depends on how efficiently the regional trends and very local (terrain) effects are removed from the gravity anomalies processed. Nettleton’s method implemented in a usual inversion scheme and combined with the simultaneous determination of terrain corrections. This method may lead to realistic density estimations of the topographical masses. The author applied this technique in the Bandar Charak (Hormozgan-Iran) with various geological/geophysical properties. These inversion results are comparable to both values obtained from density logs in the mentioned area and other methods like Fractal methods. The calculated densities are 2.4005 gr/cm3. The slightly higher differences between calculated densities and densities of the hand rock samples may be caused by the effect of sediment-filled valleys.

## Introduction

Bulk density serves as an important parameter and it is needed to interpret gravity data and determine subsurface structures. Density can be estimated from hand samples when outcrop rocks are exposed. Samples collected in the field tend to have a bias toward lower values of density because they are more weathered, less fluid-saturated, or otherwise unrepresentative of the overall density. In regions that have no exposed outcrop borehole density, logs are useful for determining subsurface densities.

The researcher determined the estimate of subsurface densities by using an inversion technique based on Nettleton (1939), and Parasnis’s (1952) which has been described in Niti (Mankhemthong et al. 2012). The author applied these techniques to analyze the gravity data. The data has been collected in the Bandar Charak area in Hormozgan- Iran. In this case, Free Air anomaly data corrected from raw observed gravity and station coordinates were considered as observed data. The obtained density estimates from the inversion method were compared to existing density data from well logs’ density and rock outcrop sample measurements. Algorithms of the proposed method were implemented via using MATLAB from Math Works, Inc.

### Geology of Bandar Charak area

**Figure 1:**
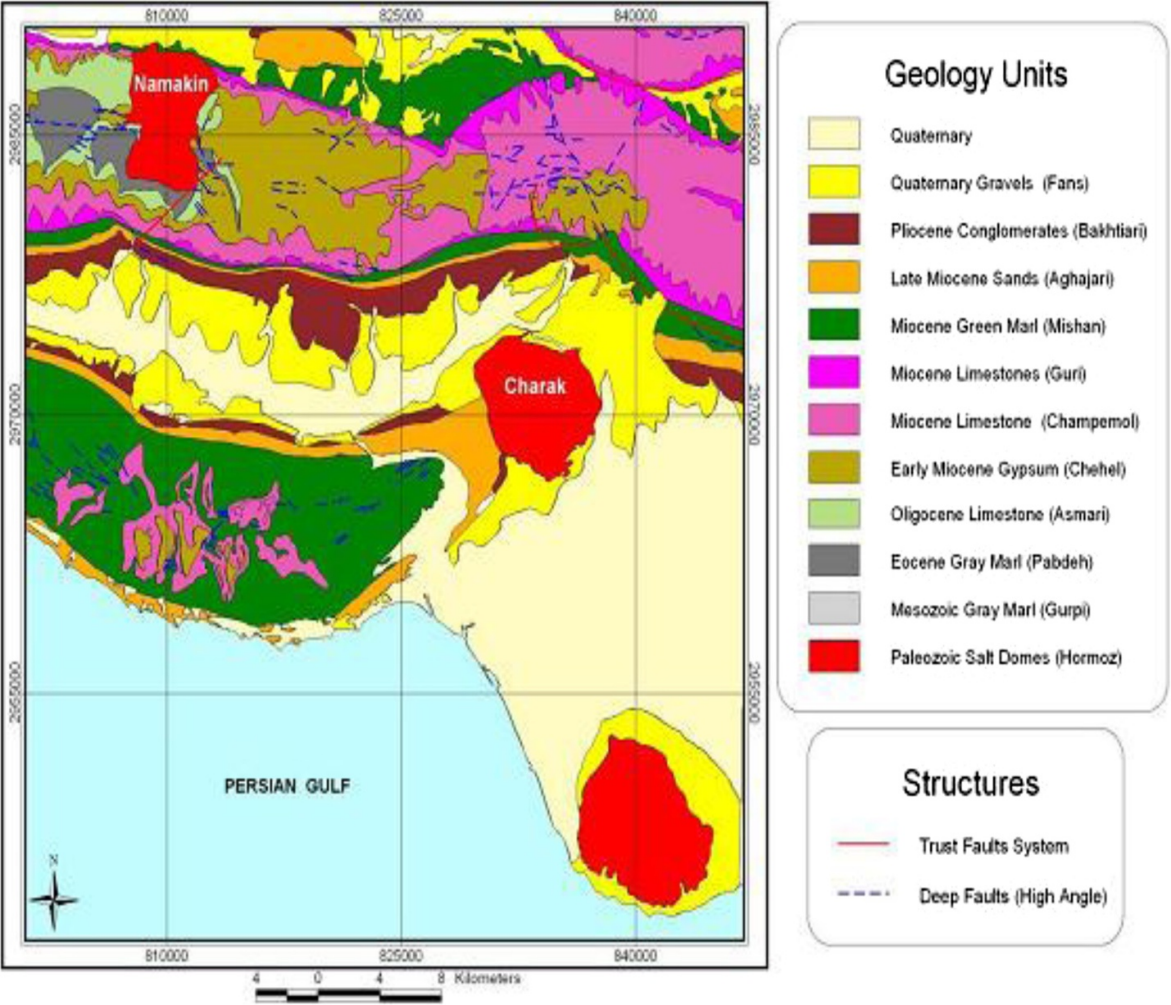
**Geological map of Charak area (National Oil Company of Iran, 2003)**

Charak area is between latitudes 27_10, 27_140, and longitudes 53_350 and 53_590. The under study area is surrounded by Ashkenan, Ahal, Boochir, Hamiran, Hashniz and Kemeshck cities. Tabnack gas structure is located in the west of this district. The area can be accessed through Asalouie (Bandar Lengeh, Lamard, and Ashkenan-Gavbandy roads). The area has a very harsh topography with mountains and valleys. The climate of the area is very hot and wet in the summer and an average climate condition in the winter. From geological point of view, Dehnow area is a part of the Fars sedimentary basin in south-east of Iran. The evidences of the salt outcrops can be recognized at two points from Dehnow anticline. Khamy formation and Bangestan group are the oldest geological structures in the area that have outcrops. Younger structures consist of Aghajary, Mokhtari, Mishan, Gachsaran and Asmary. Dominant structural trend in the area is northwest- southeast. Dehnow anticline is located between Hendurabi and Razak faults. These faults are almost perpendicular to the Dehnow anticline. Taking the combined geological-residual gravity contour map into account a northwest-southeast trend can be considered for the Dehnow anticline. A low gravity anomaly fits well the salt outcrop in the southeast of the anticline. A basic study of the geology of the area, a detail investigation of structural features such as faults associated with the Dehnow anticline, and application of the geophysical techniques, and other exploration methods is necessary to investigate the subsurface extension of the this anticline and to identify salt plug intrusion into the anticline. Gravity anomalies are the result of the interference among geological sources with different shape, densities, and depths. Of particular interest to the geologist are the linear anomalies in geophysical maps which may correspond to buried faults, contacts, and other tectonic and geological features. Most short-wavelength anomalies are caused by near-surface contacts of rocks that have different density contrasts. (Esmaeil Zadeh, et al, 2010). Table 1 show Density determination by Sampling and System Measurement in Charak region and Figure 1show Geological map of Charak area.

**Table 1:** **Density determination by sampling and system measurement in Charak region (Source: national oil company of Iran, Farmani (** 2003 **))**

Profiling no.	Coordinate (deg)		Stratum	Lithology	Density (g/cm3)
	Long.	Lat.			
L18	54°36’48.2”	26°31’48.6”	Bakhtiari Fm.	Conglomerates & Sandstone	1.87
L18					1.90
L18					1.90
L19					1.89
L19					1.86
L20	54°17’13.1”	26°47’46.0”	Mishan Fm.	Green Marl	2.14
L20					2.12
L21					2.13
L22					2.07
L22					2.14
L23	54°16’57.4”	26°48’05.9”	Aghajari Fm.	Sandstone & Marl	2.03
L24					2.02
L25					2.04
L26	53°38’17.8”	27°05’17.8”	Bangestan Grp.	Limestones	2.45
L26					2.39
L27					2.41
L28					2.45
L29					2.39
L30	53°38’18.6”	27°04’57.3”			2.44
L31					2.43
L32					2.43
L33					2.45
L34					2.44
L35	53°38’18.6”	27°04’18.3”	Asmari - Gurpi Fm.	Limestone - Gray Marl	2.36
L36					2.32
L37					2.32

### Formulations of Nettleton and parasnis density determination methods

Nettleton’s method is based on the observation that over an area of constant density no gravity anomalies should remain after applying the Bouguer correction (Papp, 2009), and that any residual Bouguer anomaly should represent the gravitational attraction of the body of interest. In the Bouguer correction formula, density value that provide the best fit of the Bouguer gravity represents the best estimate of the near surface density. Nettleton developed these methods as follow:

The relative Bouguer gravity anomaly (∆BA) between the reference station and any station is
1

Where,

BA = Gravity Bouguer anomaly

G_ob_ = Absolute gravity

G_l_ = latitude correction

G_fc_ = Free air correction (0.3086 m Gal/m)

G_bc_ = Bouguer slab correction (0.418ρ m Gal/m) where ρ is a rock density in g/cm^3^

If the correction (∆G_bc_) is ignored, Equation (1) is equivalent to the Free Air anomaly formula.
2

Where,

∆FA = Relative gravity Free Air anomaly.
3

According to Nettleton (1939), the relative Bouguer anomaly (∆BA) should go to zero if the correct subsurface density is applied during the Bouguer slab correction.
4

Where,

∆h = relative elevation change with respect to the reference station (R).

Parasnis’s method is based on the fact that the Bouguer anomaly can be expressed as an equation of the form of “y = mx + b” (Mankhemthong et al. 2012). If the region between the two stations is assumed to be homogeneous in topographic relief and density (ρ), equation (2) represents a straight line with classic form of y = mx, where the ∆FA are the y-values and 0.418∆h are the x-values. The calculated slope (m) corresponds to the average density (ρ) of the surface density rocks or sediments. The Nettleton and Parasnis methods can be used to determine near surface density if a small enough distance between gravity stations is considered. Therefore, deeper regional gravity effects do not dominate.

Rao and Murty (1973) noted that the Parasnis’s method ignored the existence of any regional gravity field. They considered the existence of uniform regional gradients in the x and y directions, with a new model where α∆x and β∆y are added to Equation (4). Here ∆x and ∆y are the distances between the gravity stations and the reference station in the x and y directions, respectively, α and β are prospered terms for unknown regional gradients that are uniform along the profiles of the two points in m Gal/km unit (Papp, 2009). After reducing the regional gravity with respect to near surface masses, it becomes:
5

∆x, ∆y, ∆h, and ∆FA are known parameters and a, b, and ρ are unknown parameters, while ρ representing the density of the subsurface. Note that Equation (5) is still a linear function of the form of “*d* = *a*_1_*x*_1_ + *a*_2_*x*_2_ + *a*_3_*x*_3_” Thus, a least squares inversion technique can be used to determine the unknown quantities.

### Development of inversion scheme

In preset study, the researcher determined estimates of subsurface densities by using an inversion technique based on Nettleton (1939) and Parasnis (1952). The last method has been described in Niti (Mankhemthong). As described in the previous section, the unknowns in Equation (5) can be determined by using a least squares inversion method. We begin by formulating the problem as
6

Where Y is the vector of reduced ∆FA, A is a matrix of perfectly known parameters containing ∆x, ∆y, and ∆h, and x represents a vector of the unknowns (α, β, and ρ).

Following the technique of Jackson (1979), Equation (6) is weighted by the diagonal matrix of the estimated covariance uncertainties (C_a_^-1^) of given free air anomalies, which are approximately 0.1-0.5 mGal.
7

Then, we multiply both side by A^T^ to begin to formulate the least squares solution.
8

Following the method of Tarantola and Valette (1982), let C_p_^-1^ be the expected covariance of the unknowns. It is assumed the covariance of the near surface density equal to the covariance of given densities from rock sample and density log measurements (~0.05 g/cm^3^). <x > is an expected unknown vector of a priori information. In an ideal situation, <x> should be equal to x.

Thus
9

And adding (10) to (11) gives;
10

Rearranging terms the below formula is obtained
11

Now x can be solved as:
12

Once the estimated unknown (x) is determined, the free air anomaly can be estimated as
13

### Inversion results

Based on the obtained results, the calculated densities were around 2.4005 g/cm^3^, while the lab measurements resulted in an average value of 2.3 g/cm3. Because of Application of Fractal methods to determine the Bouguer density in Charak region, an averaged density value equal to 2.4 g/cm3 was calculated Mehrnia et al. (2013). The result is in a good agreement with lab measurement and Fractal estimations. Moreover, from the obtained data, it is possible to mention these values:


## Conclusions

Near surface density determinations based on the Nettleton-Parasnis inversion method can be utilized for estimating representative surface densities where no outcrop or log data may exist. Densities were determined by using two methods with consistent results: (1) Nettleton’s inversion, (2) rock sampling. Based on the results, the calculated densities are around 2.4005 g/cm^3^. The greater densities from the inversion method (compared to hand samples) over the mountain loops are probably caused by the effects of un modeled topographic relief or valley fill. The calculated density uncertainties reflected the complexities of near surface lithology and structural geology beneath selected gravity stations and provided valuable information on the range of acceptable densities that can be used in further 2.5-D and 3-D forward or inverse modeling in a region.
